# Oral Angiotensin-(1-7) formulation after established elastase-induced emphysema suppresses inflammation and restores lung architecture

**DOI:** 10.3389/fphar.2025.1540475

**Published:** 2025-06-18

**Authors:** Giselle Santos Magalhaes, Alicia Villacampa, Maria Gloria Rodrigues-Machado, Maria Jose Campagnole-Santos, Robson Augusto Souza Santos, Carlos F. Sánchez-Ferrer, Concepción Peiró

**Affiliations:** ^1^ Department of Pharmacology, School of Medicine, Universidad Autónoma de Madrid, Madrid, Spain; ^2^ Vascular Pharmacology and Metabolism (FARMAVASM) group, IdiPAZ, Madrid, Spain; ^3^ Post-Graduation Program in Health Sciences, Medical Sciences Faculty of Minas Gerais, Belo Horizonte, Brazil; ^4^ Department of Physiology and Biophysics, National Institute of Science and Technology - INCT-Nanobiopharmaceutical, Biological Sciences Institute, Federal University of Minas Gerais, Belo Horizonte, Brazil

**Keywords:** chronic inflammation, cellular senescence, alveolar regeneration, Wnt/β-catenin pathway, NF-κB modulation

## Abstract

**Background:**

Chronic obstructive pulmonary disease (COPD), a prevalent age-related condition, ranks among the leading causes of global mortality. It is characterized by chronic inflammation, cellular senescence, and irreversible lung tissue damage, with no curative treatments currently available. Angiotensin-(1-7) [Ang-(1-7)] has demonstrated anti-inflammatory and regenerative potential in preclinical models. This study aimed to investigate the therapeutic effects of oral Ang-(1-7) on senescence, inflammation, and tissue regeneration in a model of elastase-induced pulmonary emphysema.

**Methods:**

Male C57BL/6 mice were subjected to emphysema induction through three intratracheal instillations of porcine pancreatic elastase (PPE). One week after the final elastase instillation, the mice were treated with Ang-(1-7) encapsulated in hydroxypropyl-β-cyclodextrin to enhance its bioavailability. The treatment was administered daily for 4 weeks. Histological assessments, gene expression analysis, and protein quantification through Western blot were performed to evaluate lung architecture, inflammation, and senescence markers.

**Results:**

The results showed that elastase exposure led to significant lung damage, including enlarged airspaces, increased collagen deposition and upregulated expression of collagen I/III and MMP9. Markers of inflammation and senescence were significantly elevated in the untreated emphysema group. However, treatment with Ang-(1-7) reversed these changes, reducing collagen deposition, restoring alveolar structure, and suppressing inflammation and senescence. Additionally, Ang-(1-7) modulated key signaling pathways, reactivating the Wnt/β-catenin pathway for tissue regeneration and inhibiting NF-κB activation, critical for inflammation suppression.

**Conclusion:**

These findings suggest that Ang-(1-7), when administered after disease establishment, demonstrates potential to reverse structural lung damage and suppress chronic inflammation in experimental models, indicating a promising direction for future translational and clinical research in COPD.

## 1 Introduction

With the global increase in life expectancy, there has been an alarming rise in the prevalence of age-related chronic diseases, including chronic obstructive pulmonary disease (COPD). COPD ranks among the leading causes of mortality worldwide and is associated with high morbidity, disability, and a relentless decline in quality of life (Divo et al., 2014; Skou et al., 2022; Agustí et al., 2023). The primary drivers of COPD progression are multifaceted, including the rampant smoking epidemic, the aging population, and, most critically, the absence of therapies that can halt or reverse the disease (Agustí et al., 2023; Huertas and Palange, 2011). Despite being both preventable and treatable, COPD remains incurable, representing a profound public health challenge (Agustí et al., 2023).

Chronic inflammation is a cornerstone of COPD pathophysiology. The initial inflammatory response could be triggered by prolonged exposure to noxious stimuli, such as cigarette smoke, the primary etiological factor in COPD. This exposure induces the migration of immune cells—neutrophils, macrophages, and lymphocytes—into the lung tissue (Huertas and Palange, 2011; Barnes, 2009; Wang et al., 2017; Wells et al., 2018). Yet, it is not solely the immune response that perpetuates this inflammatory state. Structural and resident cells of the lung, such as fibroblasts and epithelial cells, actively sustain the inflammatory cascade by producing pro-inflammatory mediators, exacerbating the chronic inflammatory response, and perpetuating it over time (Barnes, 2009).

The continuous cycle of inflammation and tissue damage in COPD results in the progressive destruction of lung parenchyma, manifesting in irreversible changes such as alveolar destruction and pulmonary emphysema (PE) (Huertas and Palange, 2011; Mannino and Buist, 2007; Cannon et al., 2016). PE is a debilitating chronic condition that imposes a substantial burden on global public health, and finding pharmacological solutions capable of reversing structural lung damage is an urgent need (Agustí et al., 2023). The imbalance between extracellular matrix synthesis and degradation impairs tissue repair, promoting unchecked lung remodeling. This cascade of events drives COPD into a state of unresolved chronic inflammation with far-reaching systemic consequences that severely deteriorate patient outcomes (Huertas and Palange, 2011; Wang et al., 2017; Wells et al., 2018; Cannon et al., 2016).

While cellular senescence is not the exclusive driver of PE and COPD progression, it plays a pivotal role in the chronic inflammatory process (Woldhuis et al., 2021; Muñoz-Espín and Serrano, 2014; Hamsanathan et al., 2019). In response to noxious stimuli and ongoing inflammation, cells undergo irreversible cell cycle arrest, entering a state of senescence (Hamsanathan et al., 2019; Tuder et al., 2012; Kheradmand et al., 2023). These senescent cells remain metabolically active, secreting a host of pro-inflammatory mediators and proteases—a phenomenon known as the senescence-associated secretory phenotype (SASP). This secretory profile amplifies inflammation through the activation of key intracellular pathways, including NF-κB, which is essential for regulating the inflammatory response (Hamsanathan et al., 2019; Tuder et al., 2012; Tsuji et al., 2006; Woldhuis et al., 2020).

Thus, in COPD, the failure to resolve inflammation induces both bronchitis and PE. In emphysema, the destruction of alveolar walls leads to reduced respiratory function, impairing gas exchange and causing air trapping, which results in lung hyperinflation (Agustí et al., 2023; Barnes, 2009; Barnes et al., 2019). This process increases respiratory effort and reduces oxygenation, contributing to systemic effects such as pulmonary hypertension and cardiovascular strain (Agustí et al., 2023). Additionally, aging exacerbates these effects, as tissue repair capacity and immune response become progressively less efficient, accelerating the structural and functional impairment of the lungs. Similarly, cigarette smoke exposure can accelerate lung aging, further worsening disease progression (Vij et al., 2018; MacNee, 2009; Ito and Barnes, 2009).

Current therapies for PE and COPD are primarily palliative, focusing on symptom management without addressing the underlying tissue damage or offering any potential for regeneration (Agustí et al., 2023). The urgent need for therapeutic strategies aimed at reversing lung damage, restoring lung architecture, and improving pulmonary function cannot be overstated. One emerging area of research that holds considerable promise is the renin-angiotensin system (RAS), specifically the axis formed by angiotensin-converting enzyme 2 (ACE2), angiotensin-(1-7) [Ang-(1-7)], and the Mas receptor (Gregório et al., 2021a; Santos et al., 2018). The considerable interest in this pathway arises from its demonstrated anti-inflammatory, anti-senescence, and pro-resolutive actions in preclinical studies (Gregório et al., 2021a; Santos et al., 2018; Magalhaes et al., 2020; Magalhães et al., 2019; Barroso et al., 2017; Bastos et al., 2020; Magalhães et al., 2015; Magalhães et al., 2020; Gregório et al., 2021b; Rodrigues-Machado et al., 2013; Magalhaes et al., 2018; Magalhães et al., 2021; Magalhães et al., 2016). Targeting the Ang-(1-7)/Mas receptor axis represents a compelling therapeutic strategy with the potential to reverse lung damage, restore pulmonary function, and arrest disease progression in PE and COPD.

In the present study, we explored the effects of an oral formulation of Ang-(1-7) on critical pathological features of PE and COPD, including senescence, inflammation, tissue remodeling, and regeneration, using an experimental model of elastase-induced pulmonary emphysema (EIPE). The findings from this investigation have the potential to drive translational clinical trials and promote a more robust collaboration between academia and industry, accelerating the development of innovative therapies to combat this debilitating disease.

## 2 Material and methods

### 2.1 Animal and ethical approval

In this study, eight-week-old male mice of the C57BL/6 strain were used, weighing between 20 and 25 g, kept in an environment with controlled light (light/dark period of 12/12 h) and fed with free access to water and food. All animal studies were performed according to national and European guidelines (2010/63/EU), approved by the Ethics Committee of Universidad Autónoma de Madrid and by Dirección General de Medio Ambiente, Comunidad de Madrid, Spain, and developed in registered animal facilities (ES280790000097 and PROEX 098.6/23).

### 2.2 Induction of pulmonary emphysema

**FIGURE 1:**
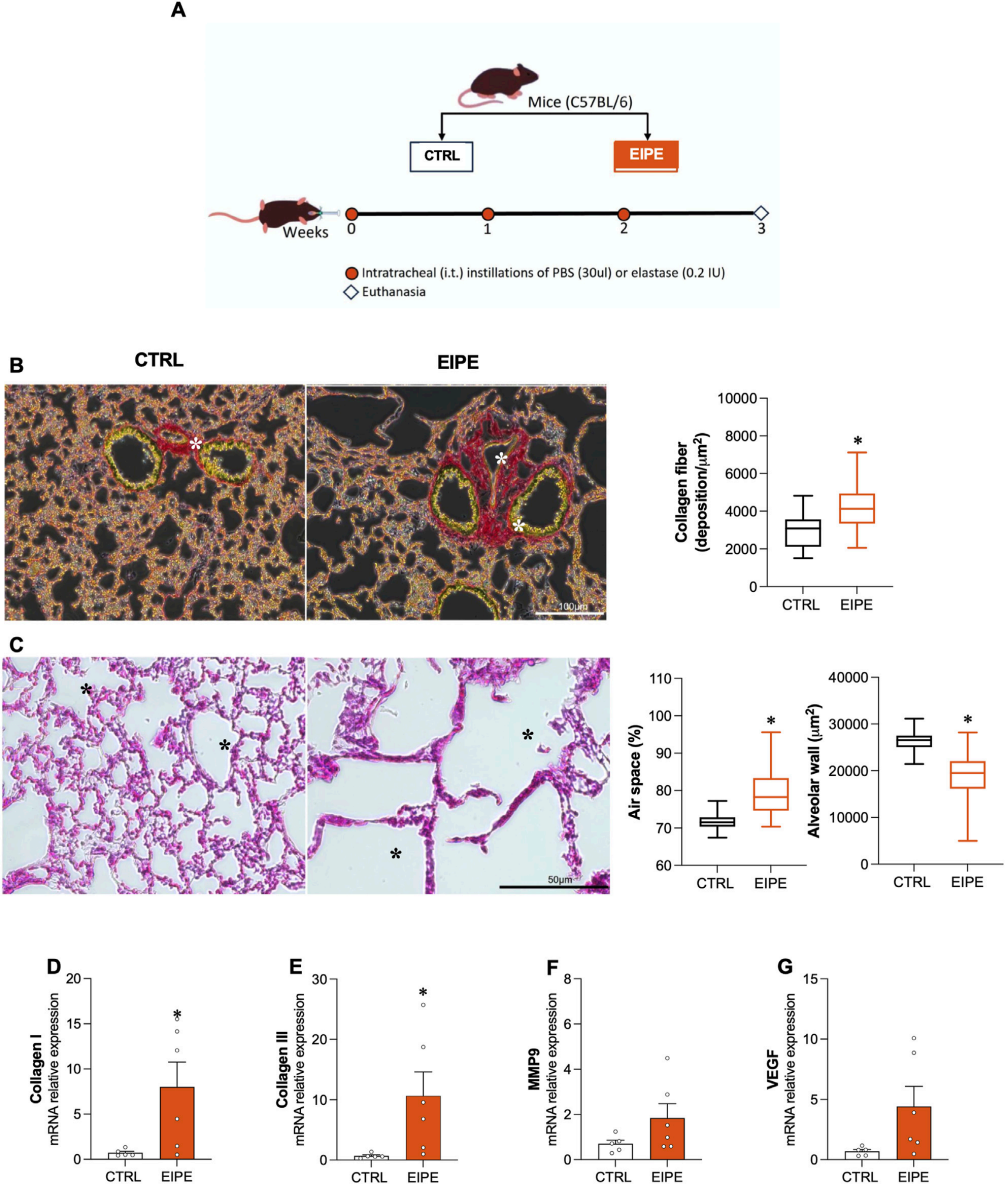
Characterization of 3 weeks evolution elastase-induced pulmonary emphysema (EIPE) Schematic timeline of the experimental design: **(A)** The pulmonary emphysema (PE) group received three intratracheal instillations of pancreatic porcine elastase (PPE) at weekly intervals (0.2 IU in 30 μL saline, n = 6). The control (CTRL) group received saline instillations at the same volume and intervals (n = 5–6). **(B)** Representative histological images of lung sections stained with picrosirius red (scale bar 100 µm) from CTRL and PE, with the graph showing quantification of collagen fibers in the lungs. As seen, elastase-challenged mice exhibited significant peribronchial and perivascular fibrosis (indicated by asterisks). **(C)** Representative histological images of lung sections stained with hematoxylin/eosin (scale bar 50 µm) from CTRL and PE. The elastase-challenged group showed increased airspace (asterisks) and reduced alveolar area compared to CTRL mice. Gene expression for **(D,E)** collagen types I and III, **(F)** MMP9, and **(G)** VEGF. Bars show mean ± SEM from four to six animals per group. *p ≤ 0.05 compared to CTRL (Student’s t-test).

The EIPE group received three intratracheal instillations of pancreatic porcine elastase at weekly intervals (0.2 IU in 30 μL saline) following the method described by Oliveira et al. (2016). The control (CTRL) group received instillations with saline in the same volume and at the same intervals. One week after the final installation, six animals from both groups (CTRL and EIPE) were euthanized, and their tissues were collected for analysis (Figure 1A). Subsequently, the remaining animals in the EIPE group were randomly assigned to two subgroups (Figure 3A) EIPE and EIPE + Ang-(1-7).

### 2.3 Ang-(1-7) treatment

Oral administration of Ang-(1-7) was facilitated by its encapsulation within a β-hydroxypropyl-cyclodextrin (HPβCD) oligosaccharide inclusion complex. This complex serves as a protective transporter, protecting the peptide from degradation during its passage through the gastrointestinal tract (Magalhaes et al., 2018). Treatment commenced 1 week following the final elastase instillation. Animals received daily oral gavage with a 100 μL solution containing HPβCD/Ang-(1-7) delivering a dose of (60 μg/kg Ang-(1-7) and 92 μg/kg HPβCD) in distilled water for 4 weeks.

### 2.4 Samples collection

Anesthesia was induced in the animals using an intraperitoneal (i.p.) injection of a ketamine (100 mg/kg) and xylazine (20 mg/kg) mixture. A midline cervical incision was made, followed by careful dissection to expose the trachea and carotid artery. The animals were euthanized by sectioning the carotid artery. Subsequently, the trachea was clamped, and the lungs were harvested at functional residual capacity. The left lung was designated for histological analysis, while the right lung was snap-frozen and stored at −80°C for further testing (Rodrigues-Machado et al., 2013).

### 2.5 Histological analysis

Following tissue collection, the left lungs were fixated in 4% paraformaldehyde and subsequently embedded in paraffin. Sections with a thickness of 4 μm were then prepared and stained with Hematoxylin and Eosin (H&E) for histopathological evaluation of the alveolar region and airspace morphology. Additionally, a separate set of sections was stained with Sirius Red to assess collagen deposition. Alveolar thickness was determined by randomly capturing 15-20 images of the alveolar region at ×40 magnification. The number of pixels between the alveolar walls was quantified in each image. Subsequently, each image was converted to binary format and the corresponding area was measured in μm^2^ (Bastos et al., 2020). The airspace was identified and quantified as the non-parenchymal and unstained area (Seimetz et al., 2011). For the assessment of peribronchial fibrosis, photomicrographs of the airways were captured under polarized light at ×20 magnification. Eight to twelve distinct peribronchial regions per lung were then selected and quantified. The results were expressed as the area of collagen deposition μm^2^ (Magalhães et al., 2020). All measurements were performed using the Image Pro Plus IPWin 4 software.

### 2.6 Western blot

Lung samples were lysed and the protein content in cell lysates was quantified by the bicinchoninic acid (BCA) method (Thermo Fisher Scientific, Illinois, United States). Subsequently, 30 µg of protein lysates were separated by SDS-PAGE electrophoresis and transferred onto polyvinylidene fluoride (PVDF) membranes (Merck, Darmstadt, Germany). Primary antibodies targeted phosphorylated p65 (p-p65; Ser536) (Cell Signaling, United States; dilution 1:1,000), p53 (sc-126; Santa Cruz Biotechnology, United States, 1:1000), and β-catenin (Anti-beta Catenin non-phospho (active) S45 antibody [EPR26155-110-1] Abcam). Following primary antibody incubation, membranes were incubated with corresponding horseradish peroxidase (HRP)-conjugated secondary antibodies (Bio-Rad; dilution 1:10,000). Protein levels were normalized to the signal from β-actin (Sigma-Aldrich; dilution 1:10,000). Immunoreactive bands were detected using an enhanced chemiluminescence (ECL) detection kit (Bio-Rad, California, United States) and quantified by densitometry software (ImageJ 1.51w).

### 2.7 mRNA isolation and quantification

Total RNA was isolated from lung samples using NZYol (NZYTech, Lisbon, Portugal) according to the manufacturer’s protocol. The concentration and integrity of the extracted RNA were subsequently evaluated using the Nanophotometer^®^ N60 (IMPLEN, München, Germany). cDNA synthesis was then performed by reverse transcription using the Maxima H Minus First Strand cDNA Synthesis Kit (K1652; Thermo Fisher Scientific; Illinois, United States) with 2 µg of RNA in a Veriti Thermal Cycler (ThermoFisher Scientific). Specific primers (Supplementary Table S1) and SYBR Green Supermix (Bio-Rad; California, United States) were employed for gene amplification in aThermo ABI QuantStudio 5 Real-Time PCR System (Thermo Fisher Scientific; Illinois, United States). The relative gene expression levels between groups were determined using the comparative CT (ΔΔCT) method with the equation 2^−ΔΔCT^. Additionally, mRNA expression was normalized to the expression of the housekeeping gene, 18S ribosomal RNA.

### 2.8 Statistical analysis

Data normality was evaluated using the Shapiro-Wilk test. Statistical significance was determined using GraphPad Prism 8 software (GraphPad Software Inc., CA, United States). All results are presented as mean ± standard error of the mean (SEM). One-way analysis of variance (ANOVA) was employed for data analysis, followed by Tukey’s *post hoc* test for multiple comparisons. The Student’s t-test was utilized for comparisons between two groups only.

## 3 Results

### 3.1 Characterization of 3 weeks evolution EIPE

This section details the characterization of the histopathological and molecular markers of lung injury caused by EIPE. After 3 weeks of elastase exposure, significant lung damage was identified, characterized by: (i) a marked increase in collagen deposition in lung tissue, particularly in the airway wall, measured by histological Sirius Red staining (Figure 1B); (ii) enlarged air spaces within the lungs, indicating the rupture of alveolar structures, further supported by a decrease in alveolar wall thickness, measured from histological H&E preparations (Figure 1C); and (iii) upregulation of collagen types I and III, determined by the enhanced production of the respective mRNAs by RT-PCR (Figures 1D,E). Additionally, the mRNA levels for metalloproteinase 9 (MMP9) and vascular endothelial growth factor (VEGF) were measured, revealing an increase that did not reach statistical significance (Figures 1F,G).

### 3.2 Characterization of inflammatory and pro-senescence markers in EIPE

**FIGURE 2:**
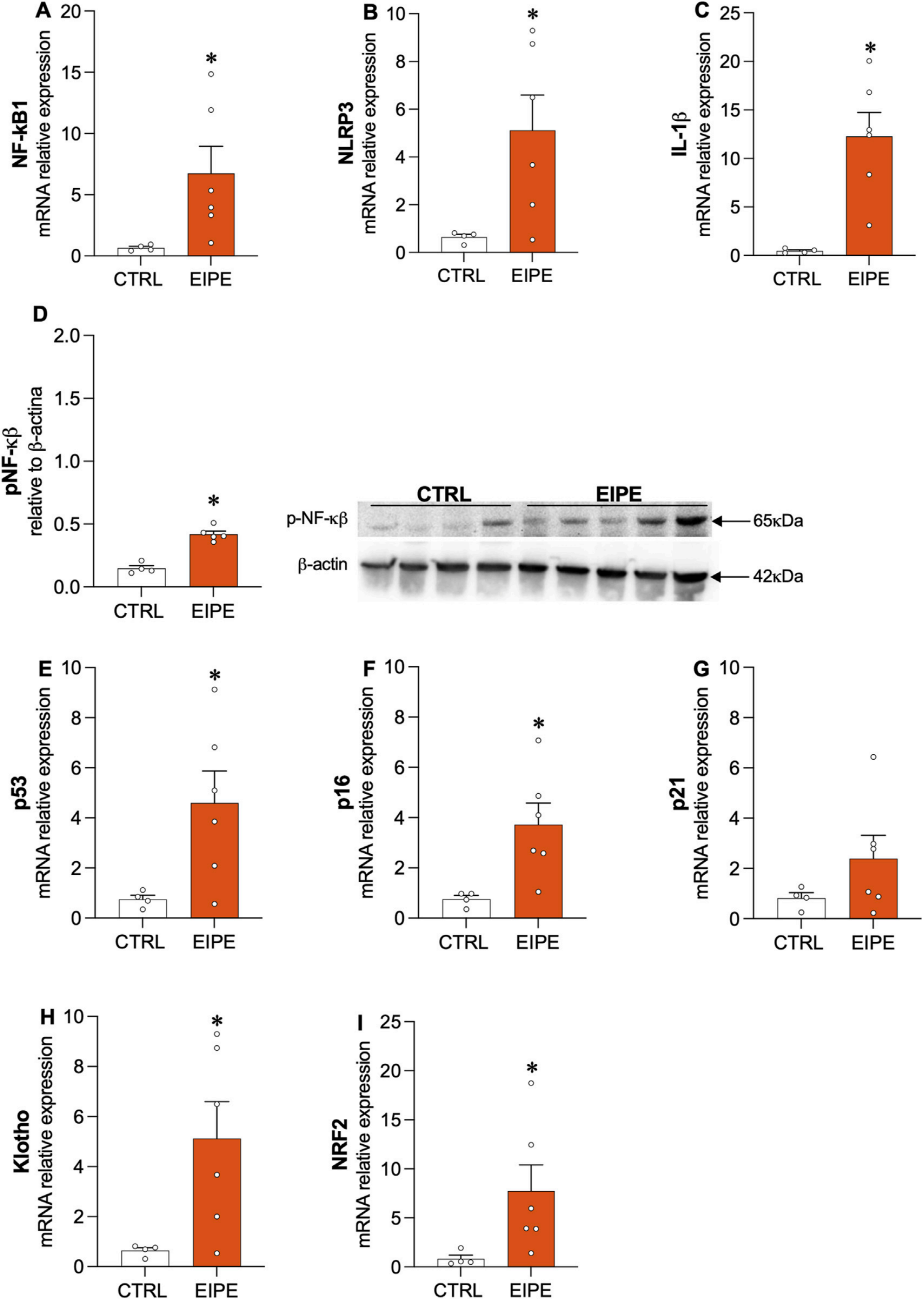
Characterization of inflammatory and pro-senescence markers in EIPE **(A–C)** Gene expression levels for inflammatory markers (n = 5–6): NF-κB1, NLRP3, and IL-1β. **(D)** Protein levels of phosphorylated NF-κB (p-NF-κB). **(E–G)** Gene expression of cellular senescence markers: p53, p16, and p21. **(H,I)** Expression of anti-senescent and antioxidant factors Klotho and Nrf2. Data are presented as mean ± SEM from four to six animals per group. *p ≤ 0.05 compared to CTRL (Student’s t-test).

Following the characterization of lung injury, Figure 2 examines key markers of inflammatory response and cellular senescence, both of which were associated with the lung damage observed after 3 weeks of EIPE progression. Specifically, genes related to the inflammatory response, including the transcription factor nuclear factor (NF)-κB1, the nucleotide-binding oligomerization domain, leucine-rich repeat, and pyrin domain-containing 3 (NLRP3), as well as the pro-inflammatory cytokine interleukin (IL)-1β, showed significantly increased expression in lung tissue following elastase challenge (Figures 2A–C). Moreover, evidence for the role of NF-κB in the lung inflammatory process was further supported by the significant increase in phosphorylated NF-κB (p-NF-κB) protein levels compared to the control group (Figure 2D). Additionally, markers of cellular senescence, such as p53 and p16, exhibited elevated gene expression after elastase exposure (Figures 2E,F), while p21 expression, although elevated, did not reach statistical significance (Figure 2G) These findings suggest the activation of inflammatory and cellular senescence pathways in response to elasFigures 2E,Ftase-induced lung injury. Interestingly, the lung tissue appeared to initiate counter-regulatory mechanisms against the heightened inflammatory and senescence signals by upregulating anti-senescent and antioxidant factors, such as klotho (Figure 2H) and nuclear factor-erythroid 2-related factor 2 (Nrf2) (Figure 2I).

### 3.3 Effects of oral Ang-(1-7) treatment on lung remodeling in EIPE

**FIGURE 3:**
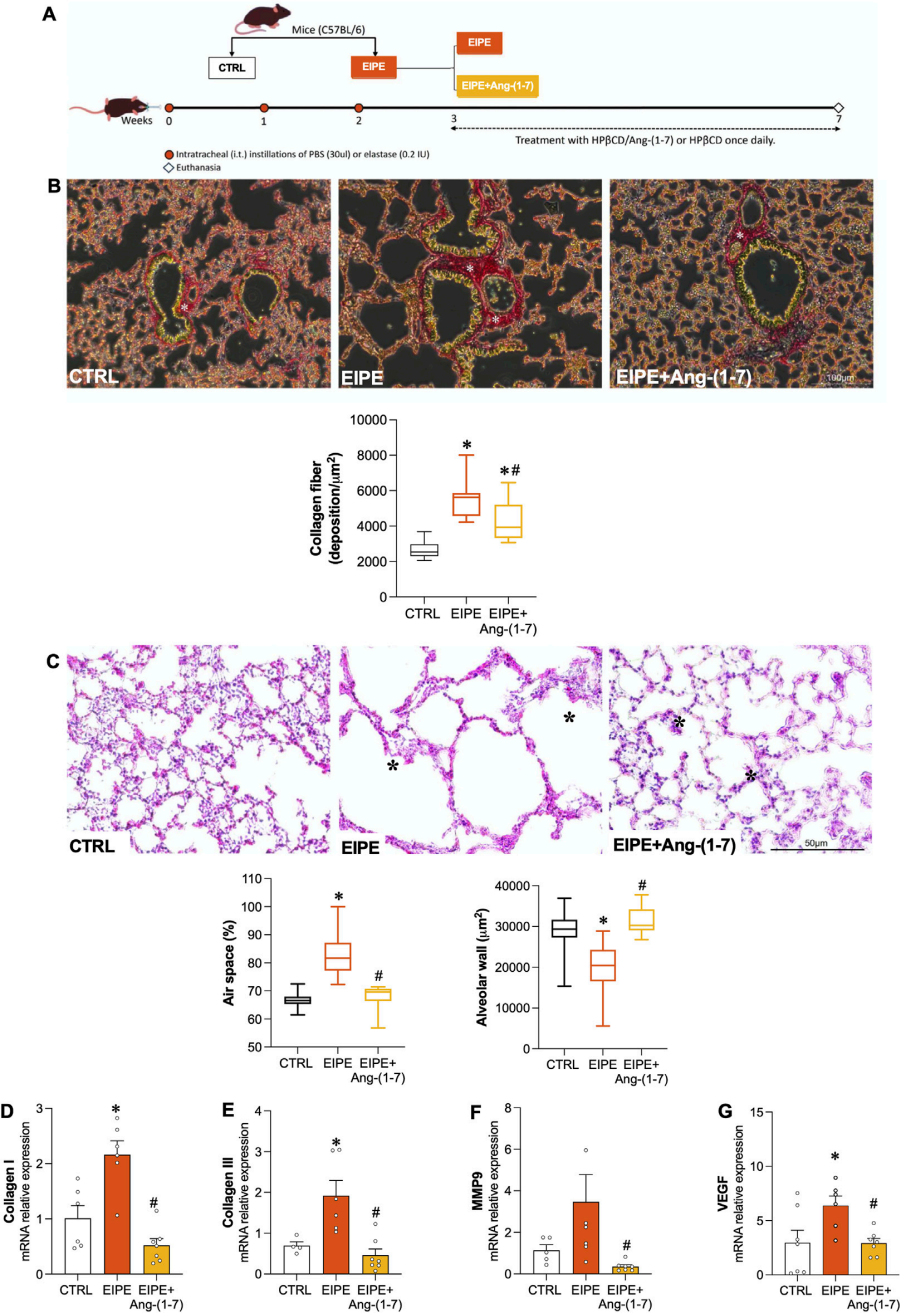
Effects of Oral Ang-(1-7) Treatment on Lung Remodeling in EIPE Schematic timeline of the experimental design: **(A)** Mice were allocated into control (CTRL) and pulmonary emphysema (PE) groups. The PE group received three weekly intratracheal elastase instillations (0.2 IU in 50 μL saline). One week after the final installation, PE mice were subdivided into PE and PE + Ang-(1-7) groups, with the latter receiving Ang-(1-7) (60 μg/kg and 92 μg/kg HPβCD) via gavage for 4 weeks. Control and PE groups received the vehicle (92 μg/kg HPβCD) via the same route (n = 5–7). **(B)** Seven weeks post-elastase exposure, the PE group showed increased lung collagen deposition (asterisk in picrosirius red-stained sections, scale bar: 100 μm). The graph represents the quantification of collagen fibers in the lungs. **(C)** Elastase exposure led to enlarged air spaces (asterisk in Hematoxylin/Eosin-stained sections, scale bar: 50 μm) and decreased alveolar area. The graph quantifies the changes in tissue area and air space percentage. **(D–G)** Gene expression levels of collagen I, collagen III, MMP9 and VEGF in the different groups. Bars represent mean ± SEM from four to six animals per group. *p ≤ 0.05 compared to CTRL, #p ≤ 0.05 compared to PE (one-way ANOVA followed by Tukey *post hoc* test).

The oral administration of Ang-(1-7) was performed on new groups of animals, as depicted in Figure 3A. After 3 weeks of elastase administration, followed by an additional 4 weeks, some EIPE mice received oral Ang-(1-7) treatment (60 μg/kg/day) to evaluate its potential therapeutic effects. Unpublished standardization results from previous studies [(Magalhães et al., 2015; Magalhaes et al., 2018)] indicated that administering Ang-(1-7) via subcutaneous, oral, intranasal, or inhalation routes does not alter the pulmonary morphological and molecular parameters in control animals.

First, we confirmed that untreated EIPE mice, after 7 weeks of disease progression, exhibited lung damage parameters that were like or even more pronounced than those observed at 3 weeks. This observation indicates that the changes in lung remodeling and alveolar damage markers in EIPE are well-established over time. Likewise, the expression of inflammation and senescence markers remained significantly elevated after an additional 4 weeks in untreated EIPE mice (Figures 3A–G), further demonstrating the chronic and progressive nature of the injury.

**FIGURE 4:**
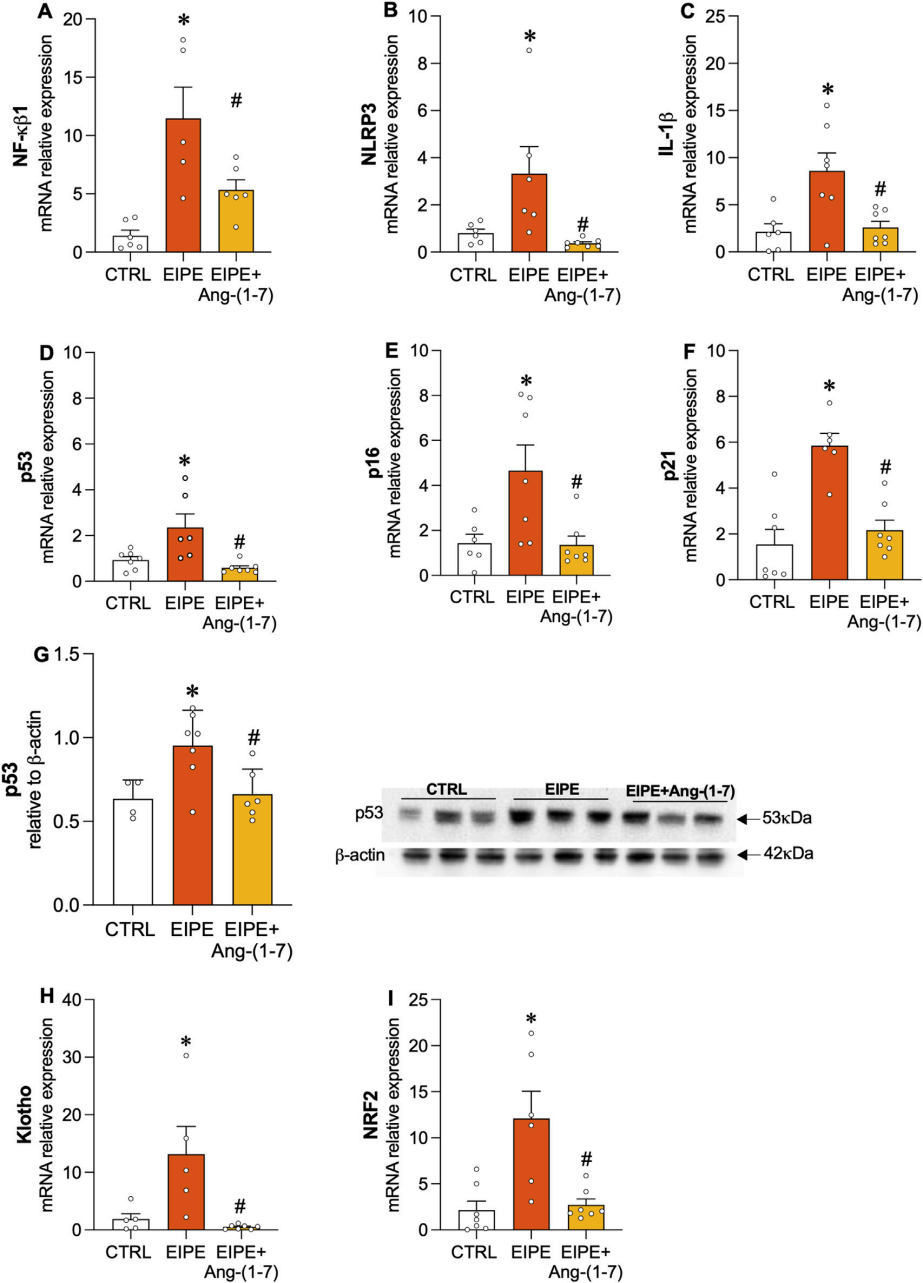
Angiotensin-(1-7) Mitigates Inflammation and Cellular Senescence in EIPE. Gene expression of inflammatory markers **(A)** NF-κB1, **(B)** NLRP3, and **(C)** IL-1β, along with senescence markers **(D)** p53, **(E)** p16, and **(F)** p21. **(G) (D)** Protein levels of p53 (n = 5–7). Gene expression of **(H)** Klotho and **(I)** Nrf2. Bars represent mean ± SEM from four to six animals per group. *p ≤ 0.05 compared to CTRL, #p ≤ 0.05 compared to PE (one-way ANOVA followed by Tukey *post hoc* test).

In contrast, oral Ang-(1-7) treatment over the 4-week period significantly reduced collagen fiber deposition (Figure 3B) and restored alveolar structure by increasing tissue area, thereby reducing the percentage of airspace (Figure 3C). Moreover, the treatment with Ang-(1-7) led to a significant decrease in mRNA levels for collagen I, III, MMP9, and VEGF (Figures 3D–G). Consistent with these findings, inflammatory markers such as NF-κB1, NLRP3, and IL-1B were significantly downregulated, as were the senescence markers p53, p16, and p21 (Figures 4A–G). On the other hand, compensatory factors that were elevated in untreated EIPE mice, such as Klotho and Nrf2, were also reduced in the animals treated with Ang-(1-7) (Figures 4H,I).

**FIGURE 5:**
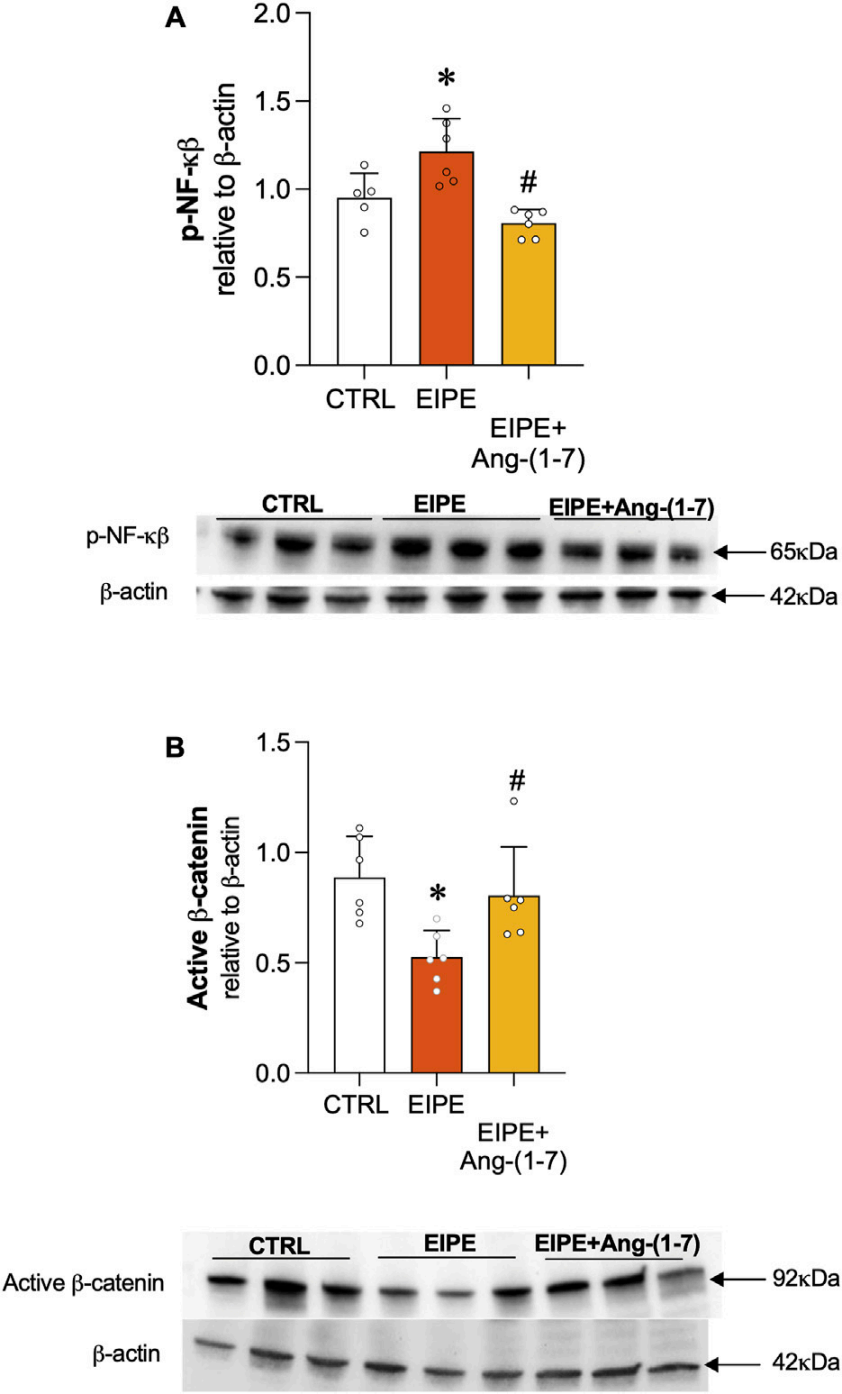
Ang-(1-7) Promotes Lung Repair by Modulating Cell Signaling Pathways in EIPE. **(A)** Expression of non-phosphorylated β-catenin and **(B)** phosphorylated NF-κB (p-NF-κB) in lung tissue after elastase exposure and treatment with Ang-(1-7). Bars represent mean ± SEM from four to six animals per group (n = 5–6). *p ≤ 0.05 compared to CTRL, #p ≤ 0.05 compared to PE (one-way ANOVA followed by Tukey *post hoc* test).

The transcription factor NF-κB is a key regulator of inflammation, often activated in response to various stressors, including those associated with lung injury. In the context of EIPE, evidence of NF-κB involvement in the inflammatory process was demonstrated by the elevated protein levels of phosphorylated NF-κB (p-NF-κB) (Figure 5A). Treatment with Ang-(1-7) significantly reduced p-NF-κB expression, highlighting its potential to suppress inflammatory pathways in elastase-induced lung injury (Figure 5A). Regarding the Wnt/β-catenin pathway, we observed a significant decrease in its expression after 7 weeks of EIPE progression (Figure 5B), aligning with previous findings that suggest this pathway is downregulated in COPD (Conlon et al., 2020). However, following 4 weeks of treatment with Ang-(1-7), the expression of active β-catenin increased to levels comparable to the CTRL group (Figure 5B).

## 4 Discussion

PE is the main pathological feature of COPD that imposes a substantial burden on global public health, and finding pharmacological solutions capable of reversing structural lung damage is an urgent need (Agustí et al., 2023). This challenge is even more critical, as COPD is more prevalent in the elderly, where both the pulmonary and extrapulmonary effects of emphysema are worsened with age (MacNee, 2009; Ito and Barnes, 2009). Aging intensifies the deterioration of lung function, including reduced respiratory capacity and chronic hypoxemia, while extrapulmonary effects, such as pulmonary hypertension, right heart failure, and muscle wasting, also become more severe (Agustí et al., 2023; MacNee, 2009; Ito and Barnes, 2009; Wei et al., 2022). Additionally, exposure to harmful agents, such as cigarette smoke, induces lung aging, accelerating the decline in respiratory function and contributing to disease progression (Tuder et al., 2012; Vij et al., 2018; MacNee, 2009). PE is a key pathological component of COPD, contributing significantly to the disease’s morbidity, particularly in elderly individuals (Agustí et al., 2023; MacNee, 2009; Ito and Barnes, 2009; Wei et al., 2022). Aging exacerbates both pulmonary and systemic manifestations of emphysema, while exposure to harmful agents like cigarette smoke accelerates lung aging and disease progression (Tuder et al., 2012; Vij et al., 2018; MacNee, 2009; Ito and Barnes, 2009). Here we utilized a robust model of PE produced by elastase to investigate the therapeutic efficacy of the Ang-(1-7) peptide in treating this disease. The EIPE model was chosen for its ability to mimic the pathophysiology of advanced human emphysema, as elastase use results in more extensive and irreversible lung lesions than cigarette smoke exposure models (Oliveira et al., 2016). This choice is justified by elastase’s capacity to cause alveolar destruction, airspace enlargement, fibrosis, and persistent inflammation—characteristics that remain present for at least 5 weeks after the final elastase instillation, reinforcing the chronic and irreversible nature of this experimental disease (Bastos et al., 2020; Oliveira et al., 2016).

Either after one- or 5-weeks following elastase instillation, we observed that inflammatory markers (NLRP3, IL-1β), senescence markers (p53, p16, p21), and the NF-κB signaling pathway remained elevated in the EIPE group, indicating that the protocol results in irreversible injury within the evaluated time frame. These findings suggest that discontinuing the elastase challenge was insufficient to induce the resolution of inflammation or tissue repair, thus establishing a chronic pulmonary damage profile. In addition to molecular alterations, significant histological changes were observed, including airspace enlargement, reduced lung parenchyma, and increased collagen deposition, underscoring the severity of the lesions caused by the EIPE model, hallmark features of emphysema progression (Barnes, 2009; Woldhuis et al., 2021; Hamsanathan et al., 2019; Tuder et al., 2012; Barnes et al., 2019; Bastos et al., 2020; Oliveira et al., 2016). The structural damage induced by elastase, including elevated levels of VEGF, MMP-9, and collagen I and III, contributes to the tissue remodeling and alveolar destruction observed in this study and others (Wang et al., 2017; Wells et al., 2018; Voelkel et al., 2007). Moreover, we noted an increase in the expression of the klotho and Nrf2 genes, two cytoprotective proteins (Hu and Moe, 2014; Li et al., 2022) known for their roles in combating oxidative stress and regulating cellular aging. This increase was interpreted as a counter-regulatory response to the exacerbated inflammatory environment induced by elastase, as previously described in chronic inflammation models. Nrf2 is expressed in the lungs, where it plays a crucial role in regulating the cellular defense against oxidative stress and inflammation (Li et al., 2022). In previous studies, Nrf2 gene deletion in mice subjected to the EIPE protocol resulted in an even more exacerbated inflammatory response, demonstrating the critical role of Nrf2 as a modulator against oxidative and inflammatory damage (Ishii et al., 2005)

In this context of severe lung injury, the mice were treated with Ang-(1-7) formulated with hydroxypropyl-β-cyclodextrin (Magalhaes et al., 2018). The treatment was administered daily for 4 weeks, and our results demonstrated that the Ang-(1-7)-treated group exhibited remarkable improvements in lung architecture, with alveolar regeneration and a significant reduction in collagen deposition. Notably, the Ang-(1-7) treatment was initiated after the establishment of emphysema, highlighting its potential as an intervention after damage has occurred. This is a critical distinction, as experimental trials with emphysema models typically initiate treatment during the challenge period (Pan et al., 2018; Zhang et al., 2018). Although Bastos and collaborators (Santos et al., 2018) began treatment with Ang-(1-7) the day after the third elastase challenge, no characterization was performed at that time to identify lung damage before treatment (Bastos et al., 2020). Thus, we provided evidence indicating the ability of Ang-(1-7) to promote lung regeneration by reducing inflammation, which suggest this agent could be a good candidate for the treatment of chronic lung diseases, even when damage has already progressed. Our studies have helped strengthen the hypothesis that the Ang-(1-7)/Mas pathway induces counterbalancing mechanisms within the RAS to attenuate pulmonary inflammatory processes (Gregório et al., 2021a; Magalhães et al., 2015; Magalhães et al., 2020; Rodrigues-Machado et al., 2013; Magalhaes et al., 2018; Magalhães et al., 2021). Furthermore, impairment of the Ang-(1-7)/Mas pathway may exacerbate the pathophysiology of inflammatory processes by preventing the resolution of inflammation (Barroso et al., 2017; Gregório et al., 2021b; Magalhães et al., 2016). With aging, there is evidence that the balance of the RAS shifts, with a relative decrease in the protective arm mediated by Ang-(1-7) (Costa-Fraga et al., 2018; Valencia et al., 2022). This reduction in Ang-(1-7) levels with aging may contribute to age-related cardiovascular and respiratory diseases, including the progression of pulmonary conditions such as emphysema.

One of the novel findings of this study is the observed effects of Ang-(1-7) on senescence markers linked to PE. Previous studies have highlighted the role of cellular senescence in chronic lung diseases, with senescent cells contributing to chronic inflammation and tissue dysfunction (Woldhuis et al., 2021; Hamsanathan et al., 2019; Barnes et al., 2019). Therefore, Ang-(1-7) ability to inhibit NLRP3 inflammasome activation and IL-1β production, along with the suppression of senescence markers such as p53, p16, and p21, suggests that the peptide possesses both anti-inflammatory and anti-senescent properties. This dual action may be particularly valuable in treating diseases like PE and COPD, where senescence and inflammation perpetuate disease progression. Indeed, NLRP3 inflammasome activation is closely linked to the maintenance of chronic hyperinflammation states characteristic of aging-related diseases, such as atherosclerosis, diabetes mellitus (González-Moro et al., 2022; Zhang et al., 2021) and COPD (Zhang et al., 2021). Previous studies have also demonstrated that activation of the Ang-(1-7)/Mas receptor axis regulates NLRP3 activation in several inflammatory models, including hepatic fibrosis (Zhang et al., 2016), neuroinflammation (Duan et al., 2021), vascular senescence (Romero et al., 2019), and pulmonary inflammation (Zhang et al., 2016; Sun et al., 2017; Huang et al., 2020).

The increase in VEGF and MMP-9, along with the exacerbated expression of collagen I and III, plays a fundamental role in the tissue damage associated with PE. Overexpression of VEGF can induce pathological angiogenesis and contribute to inappropriate extracellular matrix remodeling in lung diseases (Wang et al., 2017; Voelkel et al., 2007). Excessive MMP-9 activity leads to the destruction of elastic fibers and the alveolar matrix (Wells et al., 2018), while increased collagen I and III deposition contributes to the progressive loss of respiratory function (Agustí et al., 2023; Barnes, 2009; Barnes et al., 2019). Therefore, attenuating these markers is critical to mitigating damage and restoring tissue balance. In this context, Ang-(1-7) emerges as a promising therapeutic agent. In experimental models of pulmonary inflammation, Ang-(1-7) has been shown to suppress the production of factors that promote proliferation and fibrosis, thereby fostering an environment conducive to the resolution of inflammation and the regeneration of damaged tissue (Barroso et al., 2017; Magalhães et al., 2015; Magalhães et al., 2020; Magalhaes et al., 2018; Xu et al., 2023). Therefore, Ang-(1-7) ability to attenuate VEGF, MMP-9, and collagen I/III expression may be therapeutically relevant in the context of PE. Indeed, by suppressing these mediators, Ang-(1-7) not only interrupts the cycle of tissue degradation and remodeling but also promotes alveolar repair and regeneration.

The Wnt/β-catenin pathway plays a fundamental role in the maintenance and proliferation of type 2 alveolar progenitor cells (AT2), which are essential for alveolar regeneration (Leeman et al., 2014; Nabhan et al., 2018). Previous studies have demonstrated that Wnt/β-catenin signaling is downregulated in COPD, contributing to impaired lung repair mechanisms (Conlon et al., 2020; Uhl et al., 2015). In our EIPE model, elastase administration resulted in a decrease in β-catenin expression, indicating suppression of this repair pathway. However, treatment with Ang-(1-7) restored β-catenin expression, suggesting the reactivation of regenerative mechanisms that may be responsible for the restoration of alveolar architecture observed at the end of the treatment. One study showed that Wnt/β-catenin activation could antagonize paracrine senescence in mesenchymal stem cells, disrupting pro-senescent cascades (Lehmann et al., 2022). However, there is controversy regarding the role of β-catenin in PE, with some studies suggesting that its activation may contribute to tissue dysfunction (Lehmann et al., 2020a; Lehmann et al., 2020b). This contrast highlights the complexity of this pathway and the need for further studies to fully understand its role in different stages of lung damage. Nonetheless, the possibility that Ang-(1-7) activates a tissue repair-related pathway is a promising mechanism by which the structural damage observed in our animals may have been reversed, particularly through the activation of AT2 progenitor cells to restore alveolar architecture.

Additionally, Ang-(1-7) suppressed NF-κB activation, a major driver of chronic inflammation in emphysema (Barnes, 2009; Zhang et al., 2018). Previous studies have shown that treatment with Ang-(1-7) via different routes, such as oral and intranasal, suppresses NF-κB activation, which is directly related to inflammation suppression and reduction of leukocyte survival, thus contributing to the resolution of the inflammatory process (Barroso et al., 2017; Magalhaes et al., 2018). The interaction between Wnt/β-catenin and NF-κB pathways is crucial in PE progression, as these pathways mutually regulate each other. NF-κB activation can inhibit β-catenin signaling, impairing lung regeneration, while β-catenin activation can suppress NF-κB activity, reducing inflammation (Conlon et al., 2020; Ma and Hottiger, 2016). Ang-(1-7) dual modulation of these pathways—activating β-catenin and suppressing NF-κB—highlights its ability to reduce inflammation and enhance lung repair, positioning it as a promising therapeutic agent.

We observed a downregulation of Klotho and Nrf2 genes after Ang-(1-7) treatment, contrasting with their elevation in the untreated EIPE group. Klotho and Nrf2 are cytoprotective proteins known for their roles in combating oxidative stress and cellular aging (Li et al., 2022; Romero et al., 2019). The reduction of their levels after treatment may reflect a return to pulmonary homeostasis, with inflammation suppression and promotion of tissue repair. As our analyses were performed at the end of the 4-week treatment period, it is possible that Klotho and Nrf2 were elevated at some intermediate point, but as the tissue environment became healthier, their levels decreased—contrasting with the untreated EIPE group, which remained in a state of exacerbated inflammation.

**FIGURE 6:**
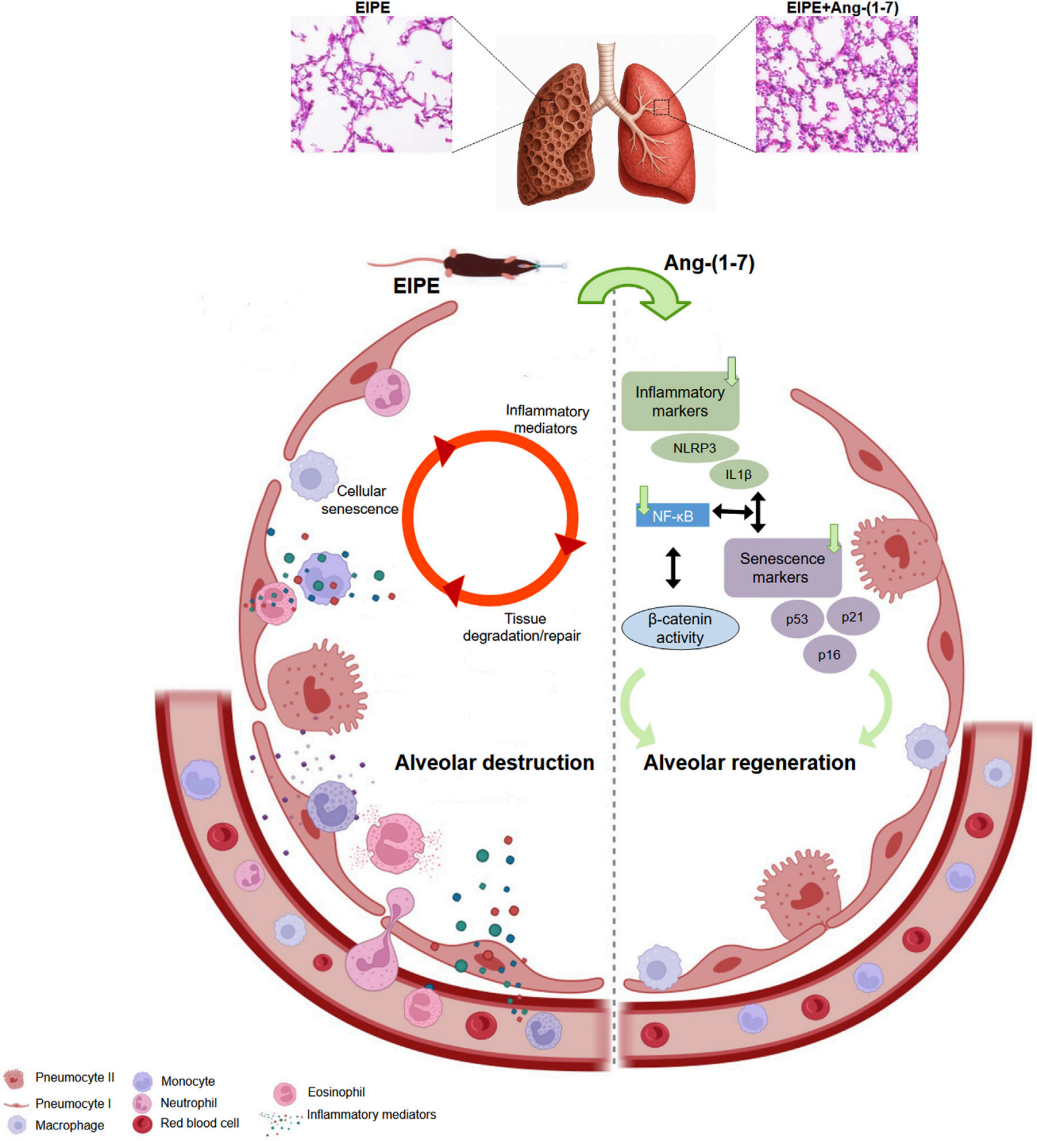
Schematic representation of the pathological mechanisms driving emphysema and the therapeutic actions of Ang-(1-7). On the left, the cycle of alveolar destruction induced by elastase is depicted, where inflammatory mediators activate senescence pathways, which in turn amplify inflammation. This vicious cycle leads to persistent tissue damage and ineffective repair. On the right, Ang-(1-7) exerts robust therapeutic effects by markedly reducing inflammatory mediators and senescence markers, suppressing NF-κB signaling, and activating the β-catenin pathway, which may contribute to the inhibition of NF-κB and plays a critical role in promoting effective alveolar regeneration. These findings highlight the powerful pro-regenerative and anti-inflammatory potential of Ang-(1-7) in the context of chronic lung injury.

In conclusion, our findings reinforce the therapeutic potential of Ang-(1-7) in treating EIPE by modulating critical pathways such as Wnt/β-catenin and NF-κB, reducing inflammation, and promoting tissue repair. Ang-(1-7)’s ability to restore alveolar architecture after injury highlights its efficacy as a post-injury intervention (Figure 6). Although our results are promising, further studies are needed to elucidate the underlying mechanisms and investigate the role of β-catenin in different stages of tissue repair, as well as the interaction between the Ang-(1-7)/Mas axis and other anti-inflammatory and pro-resolving pathways. Additionally, with aging, the reduction of Ang-(1-7) may worsen tissue damage and hinder repair. Therefore, increasing pulmonary levels of Ang-(1-7) could be a promising alternative for the treatment of chronic inflammatory diseases, especially in elderly patients. These findings provide a solid foundation for translational and clinical advancements, positioning Ang-(1-7) as a potential multifaceted therapy for chronic lung diseases.

## Data Availability

The datasets presented in this study can be found in online repositories. The names of the repository/repositories and accession number(s) can be found in the article / Supplementary Material.
