# Supplementary material for: Oral Angiotensin-(1-7) formulation after established elastase-induced emphysema suppresses inflammation and restores lung architecture
Source: Front Pharmacol. 2025 Jun 18;16:1540475. doi: 10.3389/fphar.2025.1540475 (PMC12213791; doi:10.3389/fphar.2025.1540475)
Supplement: Supplementary file 1 [file Table1.docx]

**ONLINE SUPPLEMENT**

**Oral Angiotensin-(1-7) formulation after established elastase-induced emphysema suppresses inflammation and restores lung architecture**

Giselle Santos Magalhaes^1*^, [Alicia Villacampa](https://pubmed.ncbi.nlm.nih.gov/?term=Villacampa+A&cauthor_id=38225643)^1,2^, Maria da Gloria Rodrigues-Machado^3^, Maria Jose Campagnole-Santos^4^, Robson AS Santos^4^, Carlos F. Sánchez-Ferrer^1,2*^, [Concepción Peiró](https://pubmed.ncbi.nlm.nih.gov/?term=Peir%C3%B3%20C%5BAuthor%5D)^1,2*^

^1^Department of Pharmacology, School of Medicine, Universidad Autónoma de Madrid, Madrid, Spain.

^2^Vascular Pharmacology and Metabolism (FARMAVASM) group, IdiPAZ, Madrid, Spain.

^3^Post-Graduation Program in Health Sciences, Medical Sciences Faculty of Minas Gerais, Belo Horizonte, Brazil.

^4^Department of Physiology and Biophysics, National Institute of Science and Technology - INCT-Nanobiopharmaceutical, Biological Sciences Institute, Federal University of Minas Gerais, Belo Horizonte, Brazil.

**Running title:** Ang-(1-7) reverses pulmonary emphysema.

**^*^ Corresponding authors:**

Giselle Santos Magalhaes, [giselle.santos@uam.es](mailto:giselle.santos@uam.es)

[Concepción Peiró](mailto:Concepción%20Peiró), [concha.peiro@uam.es](mailto:concha.peiro@uam.es).

Carlos F. Sánchez-Ferrer, [carlosf.sanchezferrer@uam.es](mailto:carlosf.sanchezferrer@uam.es).

Department of Pharmacology, School of Medicine, Universidad Autónoma de Madrid and Vascular Pharmacology and Metabolism (FARMAVASM) group, IdiPAZ. [C. Arzobispo Morcillo, 4, Fuencarral-El Pardo, 28029](https://www.google.com/maps/place/data=!4m2!3m1!1s0xd41858cad469ca5:0x9d8ec7a9c808c531?sa=X&ved=1t:8290&ictx=111)  Madrid, Spain. Madrid, Spain

**TABLE S1.**  Sequence of the primers used to perform qRT-PCR

| Primer | Nucleotide Sequence |
| --- | --- |
| Collagen 3 Fw  RV | GGTGGTTTTCAGTTCAGCTATGG  CTGGAAAGAAGTCTGAGGAATGC |
| Collagen 1 Fw  RV | CTTCACCTACAGCACCCTTGTG  GATGACTGTCTTGCCCCAAGTT |
| IL-1β Fw  RV | ATGGCAACTGTTCCTGAACTCAACT  CAGGACAGGTATAGATTCTTTCCTTT |
| Klotho Fw  RV | CAAAGTCTTCGGCCTTGTTC  CTCCCCAAGCAAAGTCACA |
| MMP9 Fw  RV | AGTCCGGCAGACAATCCTT  CCCTGTAATGGGCTTCCTC |
| NF-κB1 Fw  RV | GCAAACCTGGGAATACTTCATGTGACTAAG  ATAGGCAAGGTCAGAATGCACCAGAAGTCC |
| NLRP3 Fw  RV | AGAAGAGACCACGGCAGAAG  CCTTGGACCAGGTTCAGTGT |
| Nrf2 Fw  RV | AGCCTCTGTCACCAGCTCA  ATGGGGCTTTTTGATGACC |
| P53 Fw  RV | GTATTTCACCCTCAAGATCC  TGGGCATCCTTTAACTCTA |
| P21 Fw  RV | CTTGCACTCTGGTGTCTG  CTTGGAGTGATAGAAATCTGTCA |
| P16 Fw  RV | CGTACCCCGATTCAGGTGAT  TTGAGCAGAAGAGCTGCTACGT |
| 18s Fw RV | GGAAGGGCACCACCAGGAGT  TGCAGCCCCGGACATCTAA |

Fw = forward sequence; Rv = reverse sequence
